# Supplementary material for: ANTH domains within CALM, HIP1R, and Sla2 recognize ubiquitin internalization signals
Source: eLife. 2021 Nov 25;10:e72583. doi: 10.7554/eLife.72583 (PMC8648300; doi:10.7554/eLife.72583)

Source Data Map. Pashkova et al.

Fig 1B

Coomassie gel of purified ANTH domains

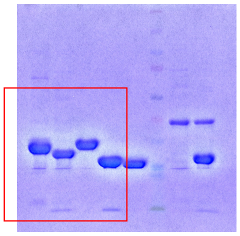

Fig 1C

GST-pulldown experiment of Hip1 ANTH domain (V5 epitope tagged) bound to GST alone, GST fused to 5 tandem copies of Ub, or a single mono-Ub.

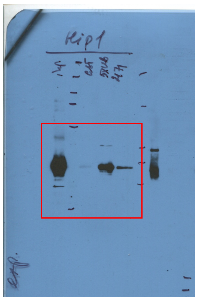

Fig. 3C

Competition experiment showing binding to Ub and Vamp7 in the presence and absence of Ub, for WT CALM ANTH domain and two mutants. Chemilumenescent immunoblot data were collected on a Flurochem instrument with a 12bit camera. Raw data were rescaled to 0-255 and inverted for display in Figure

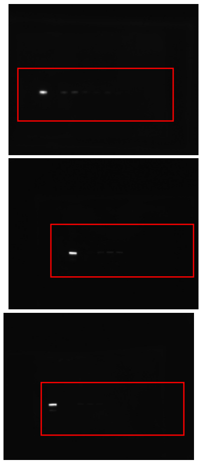

Fig. 4C

Binding experiments with immobilized GST fused to HIP1 ANTH, HIP1R ANTH domains or mutants. K63 polyubiquitin chains of different lengths were allowed to bind. After washing bound maerial as well as input was blotted with anti-Ub. Gel slices of controls and nformative mutants were used in the Figure

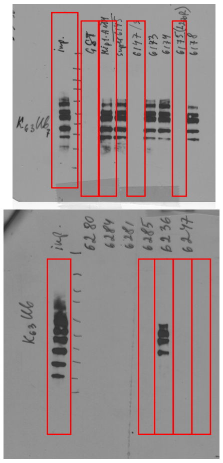

Fig. S4-1D

Yeast 2hybrid growth data of HIP1, HIP1R ANTH domains and mutants as Gal4-activation domain fusion proteins binding to Gal4 DNA binding domain fused to 2 copies of Ub. Growth of a dilution series of yeast under selective (left) and non-selective conditions.

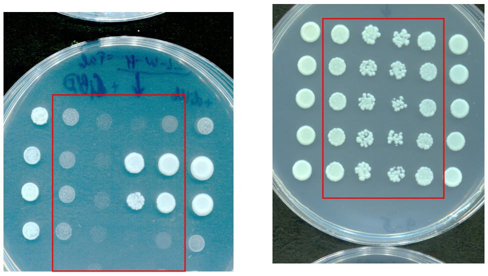

Fig. S4-1D

These screening gels were used to verify expression of the Gal4 activation domain fusion proteins used in the yeast 2 hybrid assay. Several yeast transformants and plasmids were assayed for fusion protein expression, Then the corresponding plasmids were sequenced. Gel lanes of the exact transformants used in the yeast 2 hybrid growth study are shown in red and spliced together demonstrate expression and expected molecular weight as depicted in the Figure

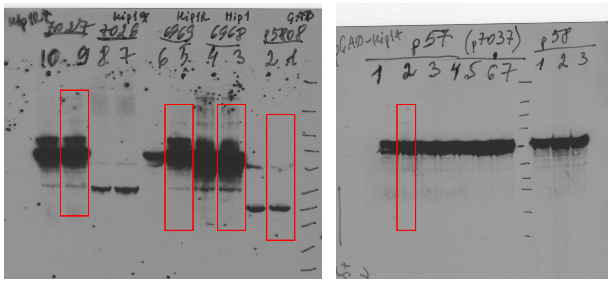

Supplement: Source data 1. — Raw images of gels and yeast plates along with a catalog of the images included. [file elife-72583-supp3.zip › SourceData/ Source_Data_Map.pdf]
