# Supplementary figures and images for: ANTH domains within CALM, HIP1R, and Sla2 recognize ubiquitin internalization signals
Source: eLife. 2021 Nov 25;10:e72583. doi: 10.7554/eLife.72583 (PMC8648300; doi:10.7554/eLife.72583)

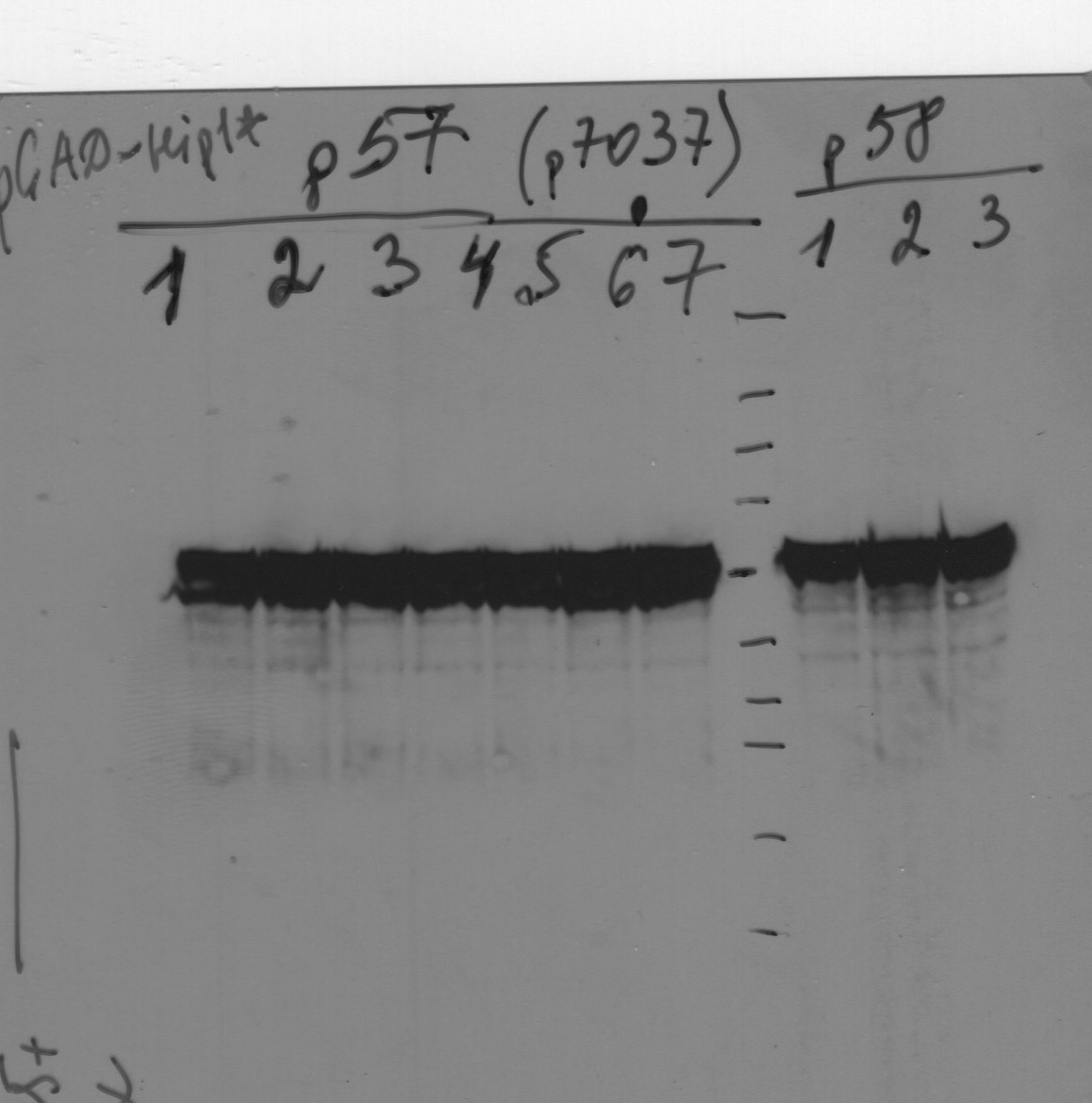

Supplement: Source data 1. — Raw images of gels and yeast plates along with a catalog of the images included. [file elife-72583-supp3.zip › SourceData/Gels and Y2H scans/SourceData_FigS4D_Blot2.tif]

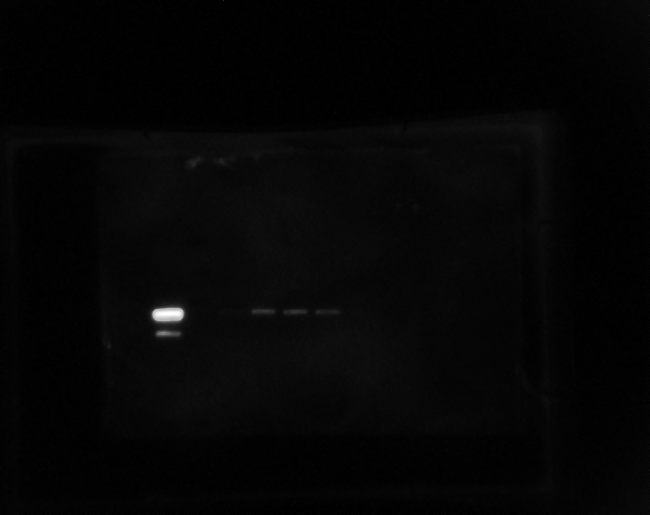

Supplement: Source data 1. — Raw images of gels and yeast plates along with a catalog of the images included. [file elife-72583-supp3.zip › SourceData/Gels and Y2H scans/SourceData_Fig3C_F223A.tif]

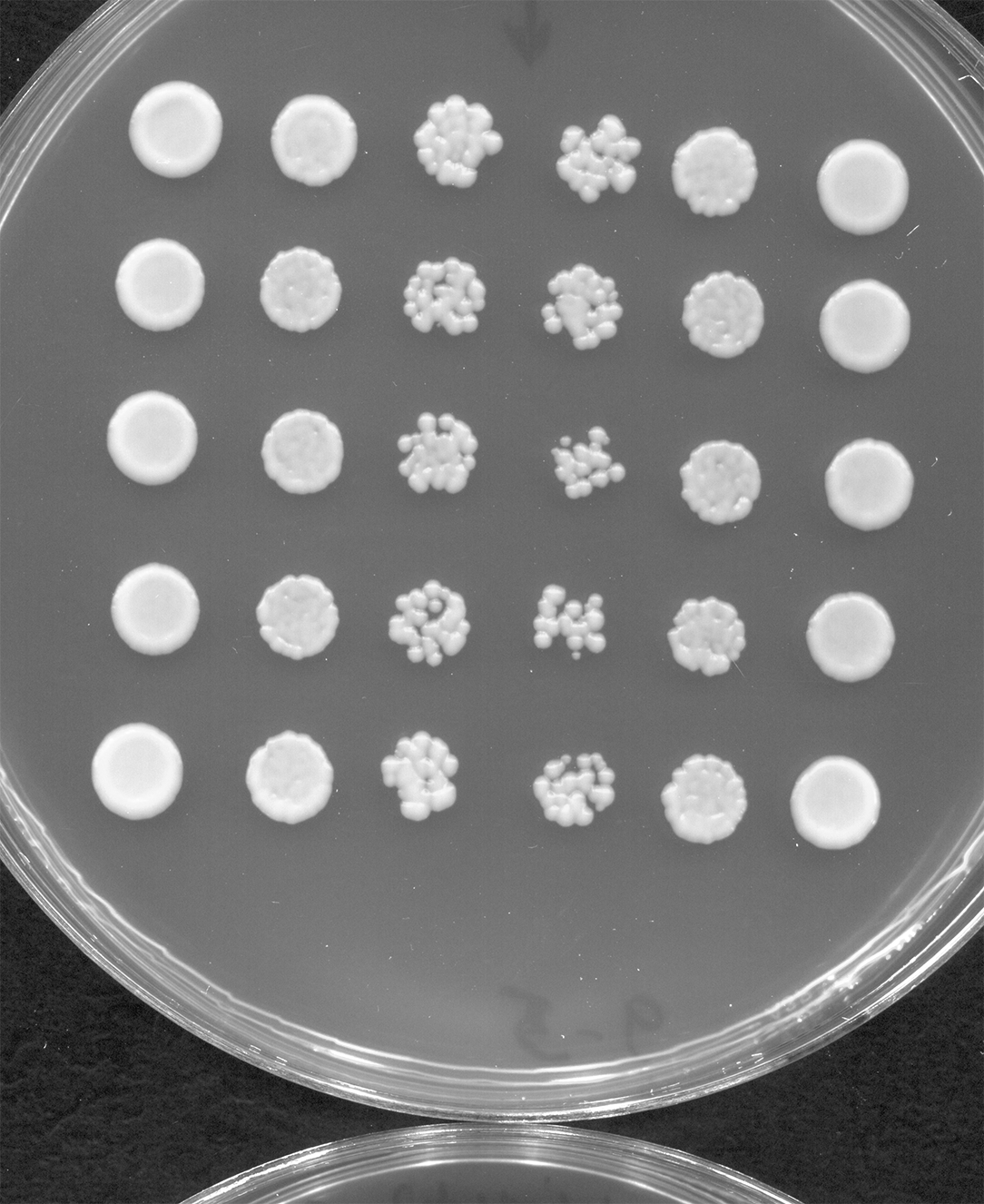

Supplement: Source data 1. — Raw images of gels and yeast plates along with a catalog of the images included. [file elife-72583-supp3.zip › SourceData/Gels and Y2H scans/SourceData_FigS4D_SD-L-W.tif]

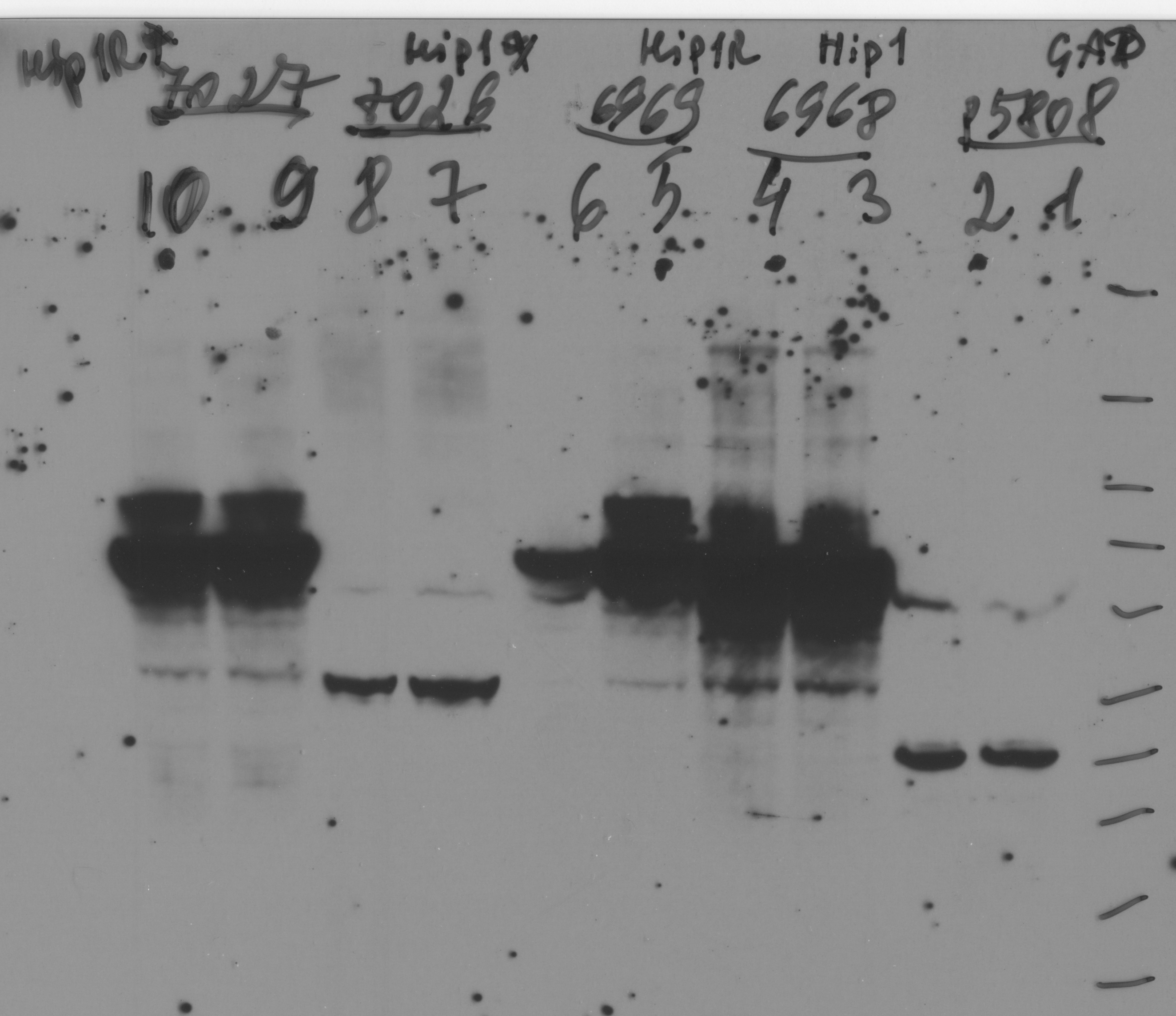

Supplement: Source data 1. — Raw images of gels and yeast plates along with a catalog of the images included. [file elife-72583-supp3.zip › SourceData/Gels and Y2H scans/SourceData_FigS4D_Blot1.tiff]

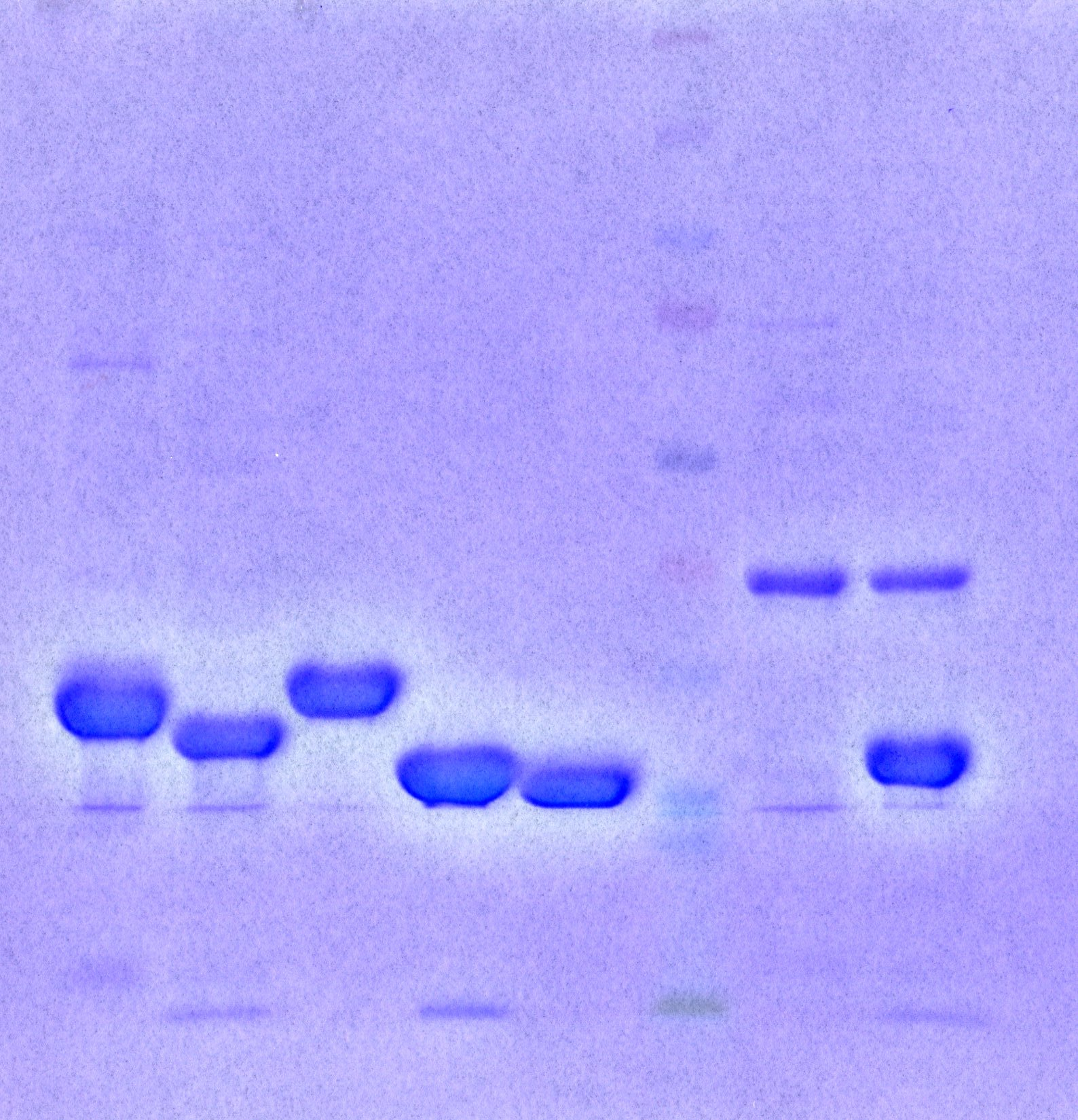

Supplement: Source data 1. — Raw images of gels and yeast plates along with a catalog of the images included. [file elife-72583-supp3.zip › SourceData/Gels and Y2H scans/SourceData_Fig1B.tiff]

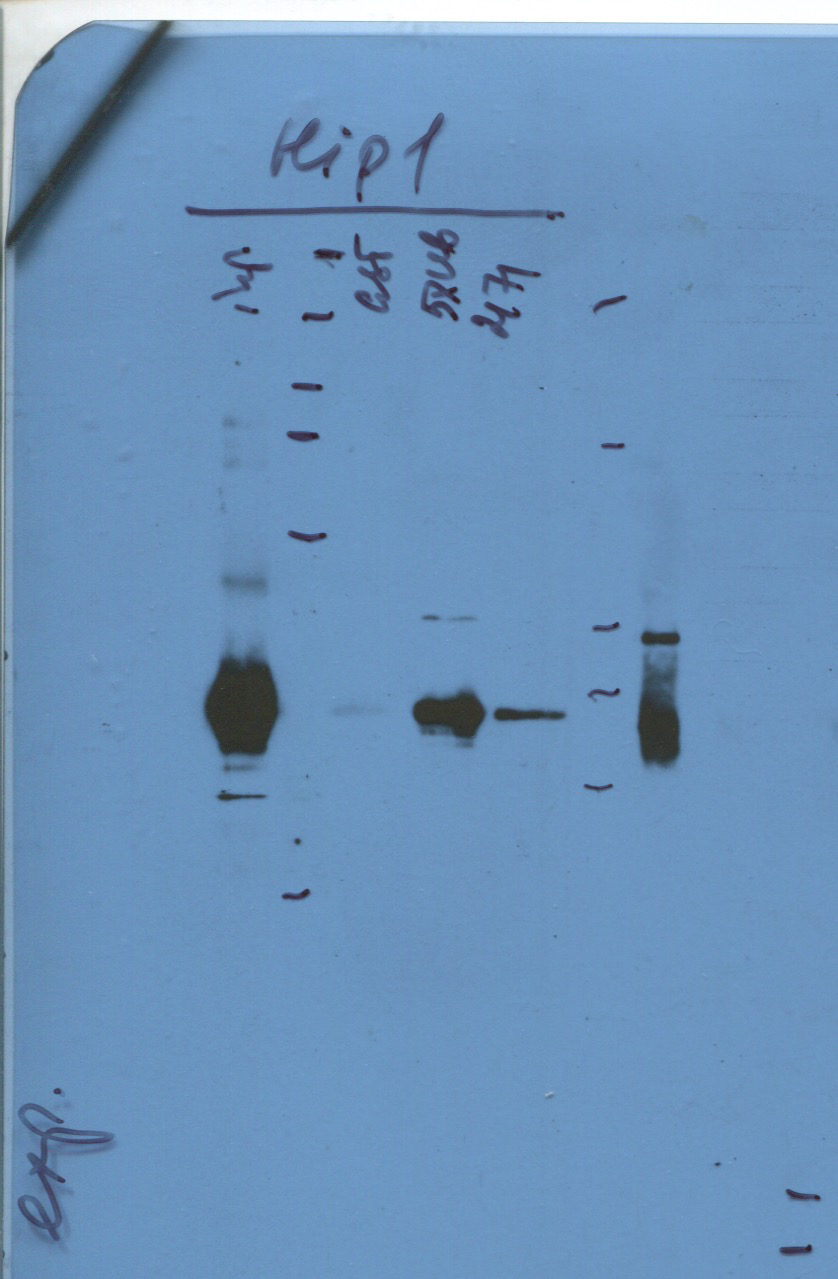

Supplement: Source data 1. — Raw images of gels and yeast plates along with a catalog of the images included. [file elife-72583-supp3.zip › SourceData/Gels and Y2H scans/SourceData_Fig1C.tif]

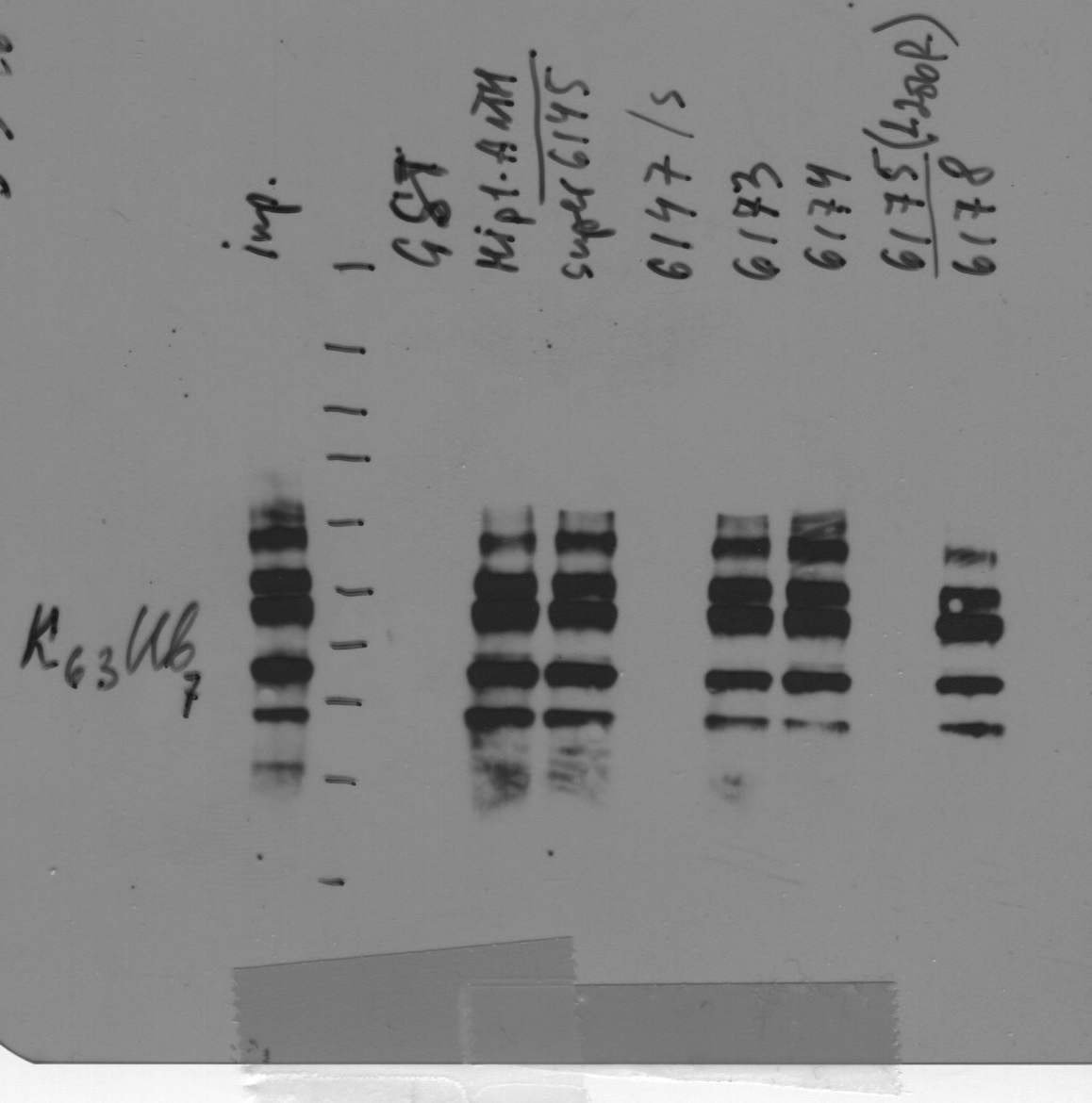

Supplement: Source data 1. — Raw images of gels and yeast plates along with a catalog of the images included. [file elife-72583-supp3.zip › SourceData/Gels and Y2H scans/SourceData_Fig4C_HIP1.tif]

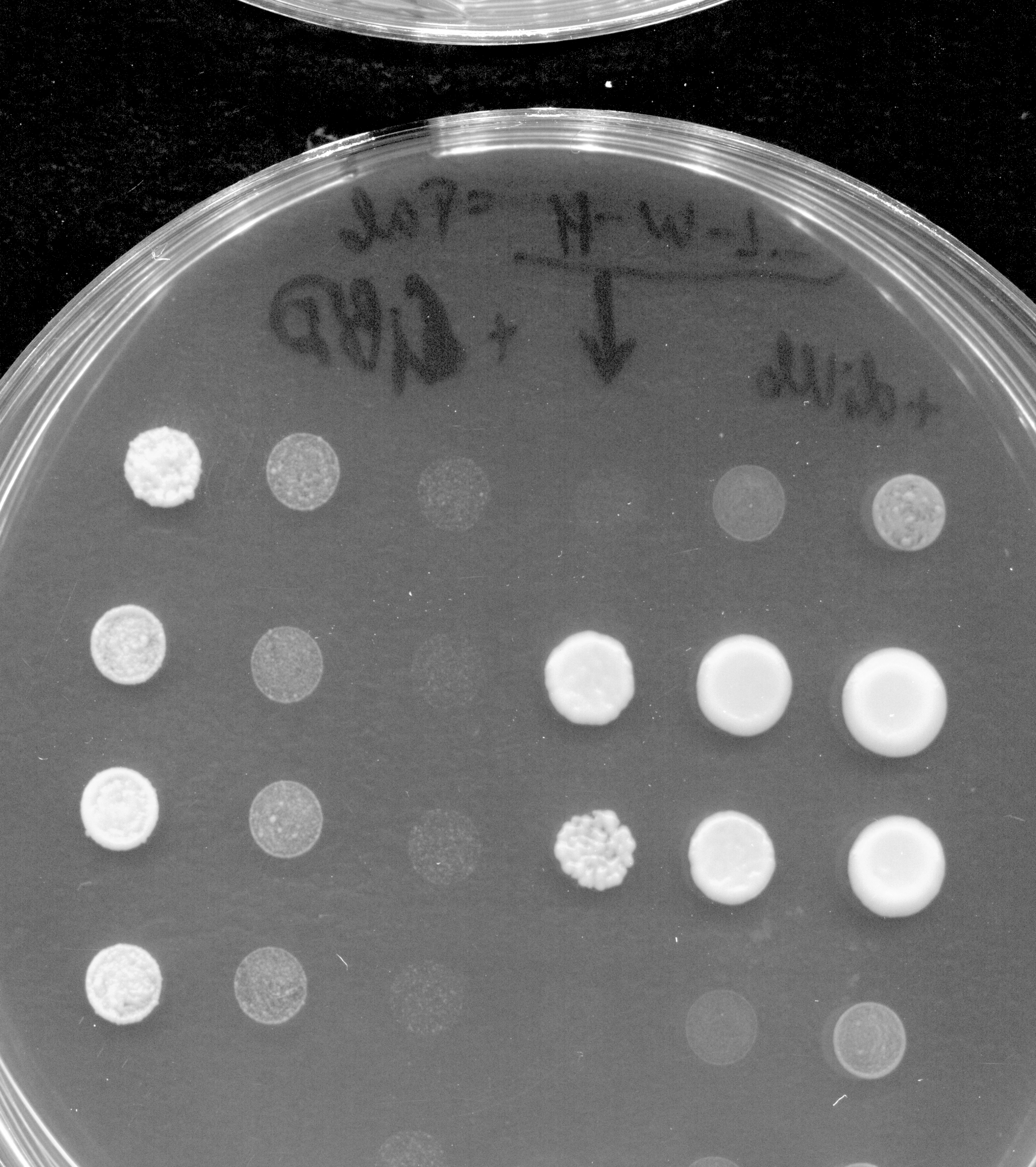

Supplement: Source data 1. — Raw images of gels and yeast plates along with a catalog of the images included. [file elife-72583-supp3.zip › SourceData/Gels and Y2H scans/SourceData_FigS4D_SD-L-W-H.tif]

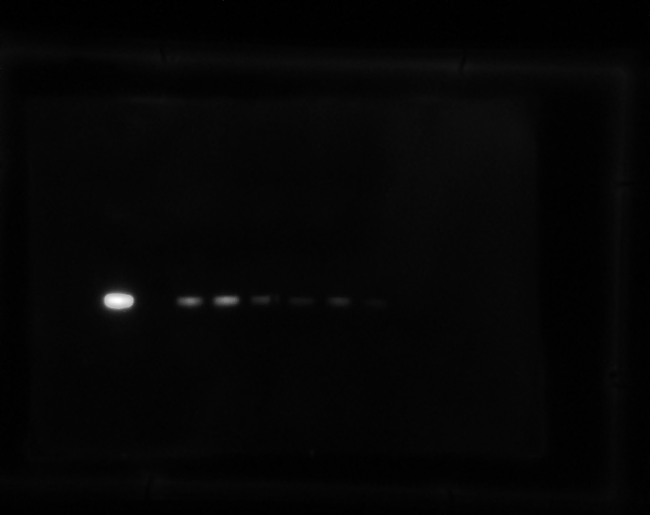

Supplement: Source data 1. — Raw images of gels and yeast plates along with a catalog of the images included. [file elife-72583-supp3.zip › SourceData/Gels and Y2H scans/SourceData_Fig3C_WT.tif]

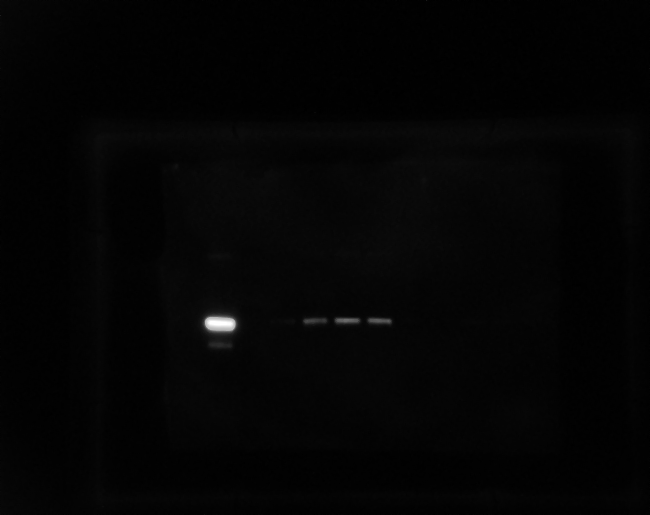

Supplement: Source data 1. — Raw images of gels and yeast plates along with a catalog of the images included. [file elife-72583-supp3.zip › SourceData/Gels and Y2H scans/SourceData_Fig3C_F223SD224R.tif]

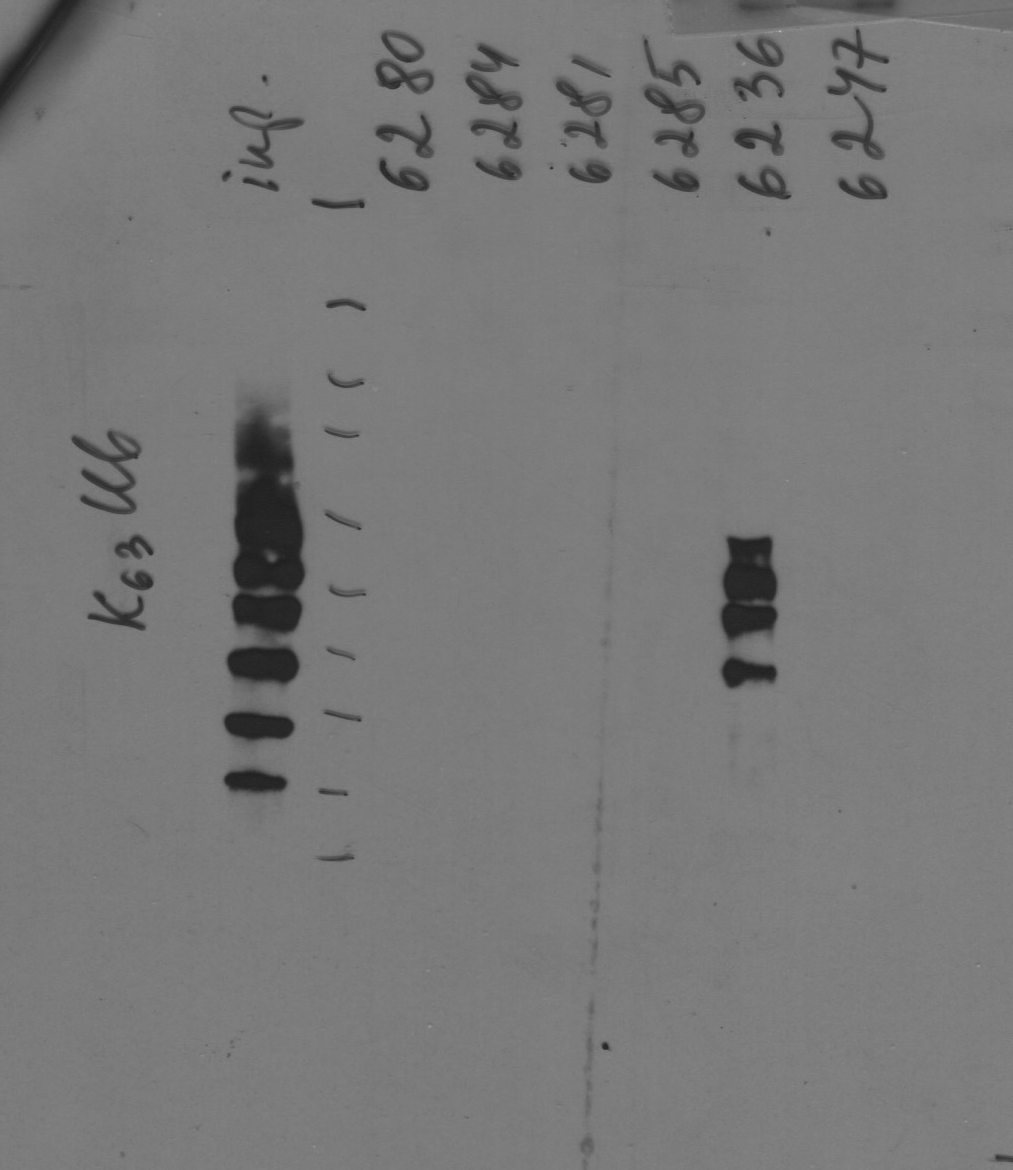

Supplement: Source data 1. — Raw images of gels and yeast plates along with a catalog of the images included. [file elife-72583-supp3.zip › SourceData/Gels and Y2H scans/SourceData_Fig4C_HIP1R.tif]
